# Reinforcement of the Maxillary First Premolar With Bayonet-Shaped Radicular Anatomy: A Challenging Case

**DOI:** 10.7759/cureus.61224

**Published:** 2024-05-28

**Authors:** Navdeep Jethi, Rachana Mishra, Charvi Gupta, Sandeep Kaur

**Affiliations:** 1 Conservative Dentistry and Endodontics, Daswani Dental College and Research Centre, Kota, IND; 2 Conservative Dentistry and Endodontics, Pacific Dental College, Udaipur, IND; 3 Conservative Dentistry and Endodontics, Guru Nanak Dev Dental College and Research Institute, Sunam, IND

**Keywords:** maxillary first premolars, prefabricated posts, bayonet shaped canals, s shaped root canal, tooth morphology, root canal anatomy, dental anatomy

## Abstract

This is a unique case of a single-rooted maxillary premolar with two separate canals in bayonet or S-shaped radicular anatomy undergoing post-endodontic reinforcement prior to crown placement. Bayonet-shaped canals present challenges in canal negotiation, cleaning, shaping, and obturation. The complexity of these canals heightens the risk of accidental file breakage and other iatrogenic errors, posing significant challenges. Post-endodontic restoration in S-shaped canals with double curvature poses challenges in precise placement and material adaptation. A novel technique was implicated to preserve the functionality and structural aesthetics of a decayed maxillary first premolar tooth.

## Introduction

Bayonet-shaped root canals, also known as S-shaped root canals, pose unique challenges during root canal treatment due to their dual curvatures in a single root [1,2]. These challenges include hindering proper instrumentation, technique-sensitive cleaning-shaping, and three-dimensional obturation, heightening the risk of procedural errors and making them one of the most intricate aspects for endodontists to navigate [3]. Ingle's research indicates that maxillary first premolars have a higher prevalence of bayonet anatomy in the canals compared to other dentitions [3]. These curvatures, whether in the mesiodistal or buccolingual directions, play a crucial role in treatment considerations, influencing the choice of instrumentation and approach taken during endodontic procedures [3,4].

Ahmed et al. further refined the Vertucci classification by identifying additional variations, including the presence of accessory canals and intricate root canal configurations based on the number of roots in these teeth [1]. Statistical data indicate that single-rooted maxillary first premolars are more common in Asian and Indian populations than those with double or triple roots [4].

These curvatures in the tooth roots are typically identified on periapical radiographs in general practice and resemble a bayonet-shaped knife, similar to those attached to rifles and used in hand-to-hand combat [5]. These curvatures exhibit two types of deviations: mesiodistal and buccolingual [5] Diagnosing mesiodistal curvatures is relatively straightforward compared to buccolingual curvatures in radiographs, as the latter often require additional imaging techniques or specialized views for accurate assessment [5]. Double curvatures can be identified by observing the shape and trajectory of the K files during canal negotiation, providing insights into root canal morphology [6,7].

Placing posts in the intricate double curvature of S-shaped radicular canals presents significant challenges during post-endodontic restorations, necessitating the application of advanced techniques and specialized instruments for achieving optimal and durable treatment outcomes [8]. This case demonstrates an innovative approach to managing challenging bayonet-shaped double curvatures during metal post placement, showcasing advancements in addressing complex root canal anatomies.

## Case presentation

Patient history

A 35-year-old female patient visited the dentist with a chief complaint of pain and food accumulation in her upper right tooth. In the last two days, the patient has felt more pain and discomfort while chewing, eating from the right side for several days, and has developed a habit of chewing only from the left side.

Clinical examination

During the clinical examination, it was observed that the maxillary right first premolar was carious, tender to percussion, and sensitive to water and air during debris removal. The electric pulp testing confirmed the presence of vital pulp. After debris removal, two canal openings were visible. Digital radiographs revealed a deep, carious lesion on the distoproximal wall of tooth 14, with radiolucency reaching the pulp. The root of the tooth exhibited double curvature, with a mesial curvature in the middle third and a distal curvature in the apical third. Multiangled radiographs failed to show the clarity of having any double roots.

Treatment plan

A root canal treatment was planned for tooth 14 with dual curvatures, requiring pre-flaring files for easier access and negotiating the distal curvature for three-dimensional biomechanical preparations. A prefabricated metal post and core buildup were required for restoration, but the rarity of such cases increased complexity.

Endodontic treatment

On the first visit, after the patient’s informed and written consent, an infraorbital nerve block was given to the patient on the right side. The tooth was isolated using a rubber dam. The remaining carious lesion was removed to healthy dentine with round burs numbers 2-4. Access was refined using an Endo Z bur (Dentsply, Bensheim, Germany). The dentinal map revealed two canal orifices in the pulp chamber: one buccal and one palatal. A size Sx Protaper (Dentsply, Tulsa, OK, USA) hand file was used to enlarge the canal orifices.

**Figure 1:**
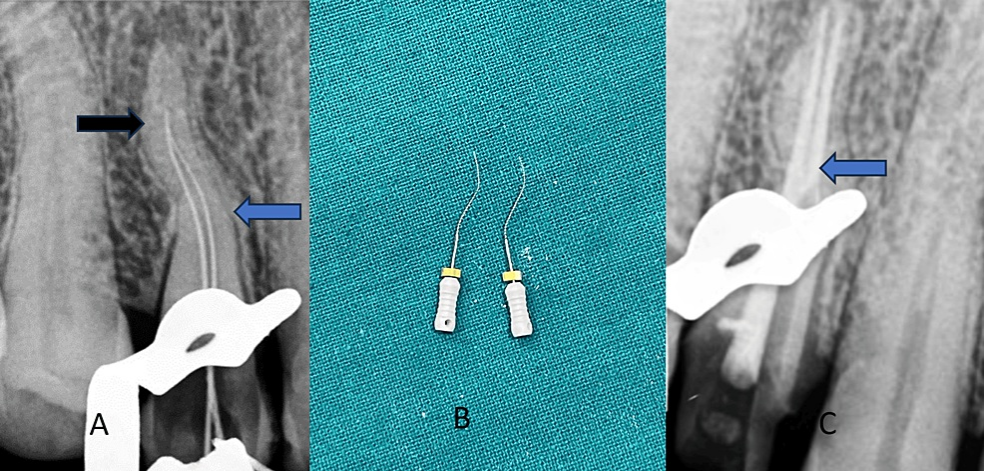
Endodontic treatment. (A) Canal negotiation curvature 1 (blue arrow) and curvature 2 (black arrow). (B) Canal patency on 15k files. (C) Sectional obturation in the palatal canal (blue arrow) and complete obturation in the buccal canal in the maxillary first premolar 14.

Canal negotiation was time-consuming and intricate (Figure 1A). The radiographs presented ambiguity regarding the identification of a single opening at the apex that was shared by both canals. Initial exploration was done using the 6k, 8k, and 10k files; pre-curving of all the files was done to navigate the curvatures. Recapitulation with a small number of files and copious irrigation with NaOCl and normal saline were mandatory to flush out the debris. After numerous attempts, the patency of the palatal and buccal canals was achieved with 15k files (Figure 1B).

Coronal flaring was done using 15k-25k files in both canals, lubricated with 17% EDTA, to get straight access to the apical distal apical curvature. Anti-curvature filing was done to scrape out the curved internal walls to straighten them for better canal shaping. Anti-curvature filing was done in two steps: first, up to the termination of the mesial curvatures of both canals, until the coronal part was almost straightened, followed by the distal curvature in the apical third. The balance-forced technique, utilizing controlled and balanced forces, was employed to shape the canal for precise and accurate treatment outcomes. The files used were 15k, 17k, 20k, 22k, and 25k hand files with an oscillating head (Orikam 1:1) on an endomotor handpiece (Dentsply) at a speed of 40 rpm with no torque, chosen for their ability to navigate the canal curvatures effectively. The 15k and 20k files (Mani, Utsunomiya, Japan) were modified by cutting 2 mm flutes to make them 17k and 22k, respectively. The small diameters of these files enhanced their ability to navigate through the curvatures effectively. Recapitulation with a smaller number of files and irrigation with every single file used were maintained throughout the procedure. Establishing the critical working length at 19 mm was crucial for ensuring precise and optimal treatment outcomes, as it was determined using an apex locator (J. Morita, Kyoto, Japan) based on radiographic measurements.

Following the 25k files, Neohybrid (NeoEndo, Orikam Healthcare, Gurugram, Haryana, India) rotary files were used with an endomotor at a speed of 500 rpm to shape the canals. Lubrication on the files and irrigation in the canal were carried out with every step.

After drying the canal using paper points, obturation was performed using Adseal (Meta Biomed, Cheongju, Korea), an epoxy resin-based sealer, and gutta-percha points (F1 size 6% taper). Sectional obturation was performed for the palatal canal, followed by complete obturation of the buccal canal using an obturation pen for vertical condensation. After a single endodontic appointment in a bayonet-shaped canal, it was temporarily sealed with an Orafil G (Prevest DenPro, Jammu, India) temporary restoration.

Post-endodontic restoration

**Figure 2:**
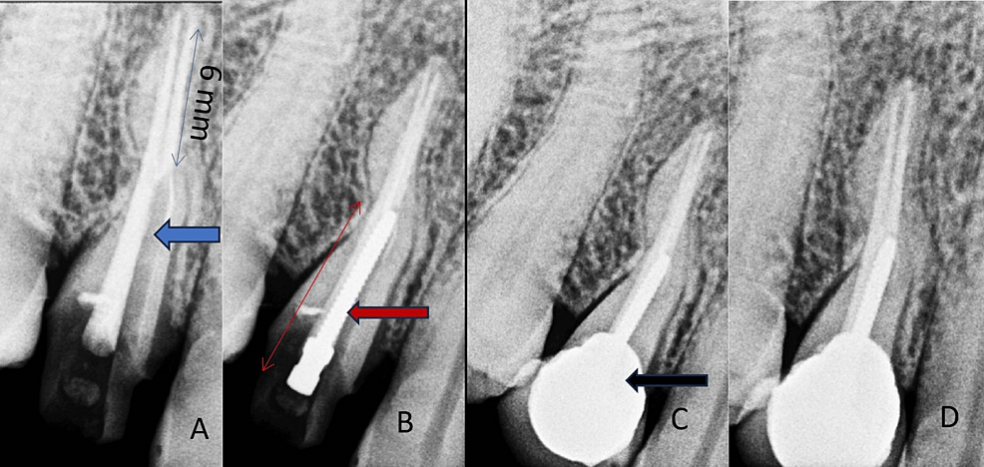
(A) Post space preparation in palatal root canal in the maxillary first premolar 14 (blue arrow), 6 mm GP left at the apex. (B) Prefabricated metal post in the maxillary first premolar 14 (red arrow). (C) PFM crown cementation in the maxillary first premolar 14 (black arrow). (D) Follow-up after six months. GP, gutta-percha; PFM, porcelain-fused metal crown

On the second visit, a post-core buildup was performed. A conservative ferrule with a chamfer marginal line was prepared on the remaining tooth structure (Figures 2A, 2B). The reduction of the coronal structure to 3 mm was necessary for optimal post-placement and core buildup. A prefabricated threaded metal post (Denext) was used. In maxillary first premolars with two canals, the palatal root is recommended for post-placement. Pesso reamer drills, sizes 1-4, were used in a crown-down manner to shape the post space (Figure 2A). Due to the double radicular curvature of bayonet-shaped anatomy, both curvatures were not included in the post space and were terminated up to the beginning of mesial curvature. It is recommended to leave the curvatures of a canal untouched, as per Ingle; 6 mm of the gutta-percha was retained at the apex (Figure 2A). Also, 1 mm of healthy root canal dentine was maintained on the lateral walls of the entire diameter of the post space in the canal. The post diameter used was 0.7 mm for the prefabricated threaded metal post. The post-fit was checked (Figure 2B, Table 1).

**Table 1:** Considerations for post placement in S-shaped teeth with two roots or two root canals

Selection of root or canal for the post	Palatal root or root canal
Remaining gutta-percha at apex	5-6 mm
Post termination	Up to the beginning of the first curvature
Curvatures	Should not be included in the post space
Root canal dentine thickness	Minimum 1 mm on the entire circumference of the root canal
Post diameter	Less than 0.7 mm

A resin-based cement was applied to the post space, and the metal post was carefully screwed clockwise into the dentine using a post key for secure placement. After post-placement, core buildup was performed using composites (Tokuyama Estelite, Tokyo, Japan). A chamfer line was achieved on the core using a tapered round bur in the air rotor. An alginate impression was taken. On the third visit, a porcelain-fused metal crown was cemented to this core (Figure 2C).

Follow-up

The six-month follow-up revealed good functional and structural retention in tooth 14, with no signs of periapical infection, and the patient reported satisfaction with the treatment outcomes. An intraoral periapical radiograph was taken, revealing no signs of periapical infection during the follow-up assessment. The patient expressed great satisfaction and relief with the absence of any pain following the treatment (Figure 2D).

## Discussion

In this case report, the observation of S-shaped anatomy with double curvatures in the two canals of the single-rooted right maxillary first premolar presented challenges in achieving accurate radiographic documentation due to the intricate and complex nature of these curvatures. Also, treating a double-curvature tooth posed a challenge due to the rarity of previous instances where such cases were treated or documented, leading to increased complexity in treatment planning and execution.

Maxillary first premolars exhibit significant variations in root anatomy and morphology, making them often unpredictable [2]. According to the Vertucci classification, maxillary first premolars can have all eight variations of pulp chamber configurations, including those with one or two canals [2,3,8]. This classification was further refined by Ahmed et al., who identified additional variations based on the number of roots present in these teeth [1]. Moreover, single-rooted maxillary first premolars have a higher prevalence in the Indian population compared to those with double or triple roots [4].

According to Ingle, maxillary first premolars exhibit a higher prevalence of bayonet anatomy in the canals, with a rate significantly greater than that found in other dentitions [1,3,7]. Mesiodistal curvatures account for 38.8% of the curvature in these premolars, while straight (30.9%), buccopalatal (27.6%), and S-shaped curvatures are very uncommon (2.7%) [9]. According to research by Yan et al., 94.2% of the maxillary second premolars in a Western Chinese population with 1,118 cone beam CT scans revealed that 55.1% of the teeth had a single canal [10].

In this case, a post and core buildup were essential to ensuring the longevity of function and strength in the double-curved maxillary premolar. There is a scarcity of literature on the post-endodontic restoration of a metal post in bayonet-shaped canals.

Utilizing the oscillating head with the K files not only facilitates faster cleaning and negotiation of the canals but also eliminates the need for torque, contributing to the procedure's precision [11-13]. Implementing the file modification technique was crucial to addressing narrow canals and improving the flexibility and adaptability of the files during negotiation [7].

In this case, both curvatures were intentionally excluded from the post space to reduce the risk of vertical fractures associated with including tooth curvatures. The sectional obturation was accurate up to 6 mm at the beginning of the first curvature. Root curvatures significantly influence post-termination and retention in canals with complex anatomies, crucially impacting the success of post-placement [2,3]. As per Ingle, if there is 5 mm of gutta-percha remaining at the apex after post space preparation, it is advised to leave the curvatures in the roots undisturbed [3]. It is recommended to terminate the post length where the curvature begins to avoid root perforation, iatrogenic errors, and damage to the remaining root dentine, ultimately improving fracture resistance [3]. It is recommended to use a post diameter of less than 0.7 mm for premolars to maintain a 1-mm lateral dentin thickness, ensuring adequate structural support and minimizing the risk of complications [3].

The intricate root anatomy, with canal structures such as bayonet or S-shaped canals, creates challenges in achieving precise post-endodontic reinforcement and accurate crown placement and may lead to potential complications or treatment failure [3,8]. Furthermore, the uncertainty surrounding the long-term success and durability of reinforcement in a complex root structure is attributed to the intricate nature of the canal system, including the presence of two curves and separate canals in bayonet or S-shaped configurations [3].

## Conclusions

During post space preparation in bayonet-shaped canals, it is crucial to exclude the curvature in post-termination to ensure accurate and successful post-placement. Reinforcing bayonet-shaped root canals after endodontic treatment continues to be a significant challenge. The pronounced dual curvature and the necessity for precise techniques and specialized materials, such as specific posts and sealers, are required to ensure successful reinforcement.
